# Insights into handling and delivery of Y-90 radioembolization therapies

**DOI:** 10.3389/fnume.2023.1075782

**Published:** 2023-03-21

**Authors:** Dustin R. Osborne, Gregory Minwell, Bradley Pollard, Chris Walker, Shelley N. Acuff, Kristen Smith, Cain Green, Rachel Taylor, Christopher D. Stephens

**Affiliations:** Department of Radiology, University of Tennessee Graduate School of Medicine, Knoxville, TN, United States

**Keywords:** Y90, radioembolization, radiation safety, Yttrium-90, PET/CT, residual dose, dosimetry, therapy

## Abstract

**Introduction:**

The use of Y-90 radioembolization techniques has become a standard tool for the treatment of liver cancer and metastatic diseases that result in liver lesions. As there are only two approved forms of radioembolization therapy, the procedures for use are also fairly standardized even though exact international and interdepartmental procedures can vary. What has been less published over the years are the nuanced differences in delivery techniques and handling of the two available Y90 radioembolization therapies. This paper seeks to examine various aspects of delivery techniques, product handling, and radiation exposure that differ between the available and approved products. Understanding these differences can assist with providing more efficient treatment, confirmation of accurate therapy, more informed handling of the products, and improved training of physicians and other hospital staff.

**Methods:**

Two commercially available and approved radioembolization devices were compared to assess nuanced, but key differences between the available products regarding therapy delivery, handling of the products, and radiation exposure to patients and staff. This work is broken into two sections: (1) Therapy Delivery, (2) Radiation Safety. Therapy delivery characteristics were assessed by using an external radiation detector system with detectors placed inside of each delivery system facing the dose vial and on the output catheter lines to the patient. Additional detectors were placed near the liver of the patient and on top of the foot to measure extremities. Data were acquired continuously throughout therapy delivery to collect time activity curves (TACs) for the characterization of each therapy. These data were analyzed to assess if (a) real-time monitoring of radiation could be used to provide an accurate assessment of residual dose before the patient leaves the procedure room, and (b) can dose delivery characteristics be observed that enable improved training and quality control. Calculation of residual dose using the external detector TACs was performed by analyzing initial and final activity peaks to determine measured count rate differences. Radiation safety aspects were assessed by monitoring radiation exposure to staff handling each of the available therapy products. Nuclear medicine technologists and interventional radiology physician body and hand doses were measured for each delivered therapy using standard body and ring dosimeters. The TACs noted above collected for the liver and extremities were used to assess if any off-target or leached Y90 activity could be detected for each therapy. Blood was collected at times before, during, and after treatment and then counted on a gamma counter to assess differences in free Y90 circulating in the blood. Each patient in this study also received a post-treatment whole-body PET/CT at 2–4 h post-infusion to assess for any aggregate free Y90 deposition that may have resulted from circulating free Y90 in the subject following therapy.

**Results:**

Calculations of residual dose in the vial following therapy using the real-time detection methods resulted in values that were not statistically different from the values calculated by nuclear medicine following the procedure (p>0.05). Real-time collection of dose delivery data enabled observation of key characteristics related to each delivery method. For SIR-spheres procedures, the cycle of pushing the dose and visualizing with fluoro can easily be seen with each push resulting in a smaller and smaller peak with intermittent fluoroscopy pulses. TheraSpheres infusions show a rapid bolus with nearly all of the measurable injected activity being infused in the first push of the dose. Staff radiation exposure assessments showed statistically significant differences between glass and resin spheres for hand doses of physicians and technologists (*p* > 0.05), but no statistical difference between body doses for both products (p>0.05). Assessments of free Y90 circulating during therapy showed that patients undergoing therapies with resin spheres had post-infusion blood levels that were 120% higher than pre-infusion levels while glass sphere therapy patients only saw a 7% rise in post-infusion blood levels. The coefficients of variation (COVs) across glass sphere measurements pre, during, and post, were only 0.008 while resin sphere measures saw much greater variability with a COV of 0.45. Both glass and resin therapies showed blood levels at 2–4 h post-injection to be similar to levels measured pre-injection. Neither therapy showed any signs of focal aggregation at 2–4 h post-infusion on whole-body PET/CT.

**Conclusion:**

Although glass and resin radioembolization therapies are similar, they both have unique characteristics related to their administration and handling by staff. Understanding the nuances can assist in providing more efficient delivery, better staff education, and reducing radiation exposure to everyone involved with these therapies. The use of near real-time monitoring is feasible and can be used to obtain critical information about the delivery success of a therapy and can inform physicians on their techniques to optimize their practice as well as provide more consistent training to residents.

## 1. Introduction

Hepatocellular carcinoma (HCC) accounts for nearly 90% of all primary liver cancers and represents more than 815,000 cases annually worldwide (1–6). When you factor in additional cancers that can metastasize with liver-dominant diseases, such as colorectal cancer, the numbers are even more significant (7). This has led to a high prevalence of the use of Y90 therapies in hospitals that can support the necessary infrastructure. These types of procedures, although not without risk, have shown excellent outcomes for patients with median 1 and 2-year survival rates being above 80% and 65%, respectively (8,9).

**Figure 1:**
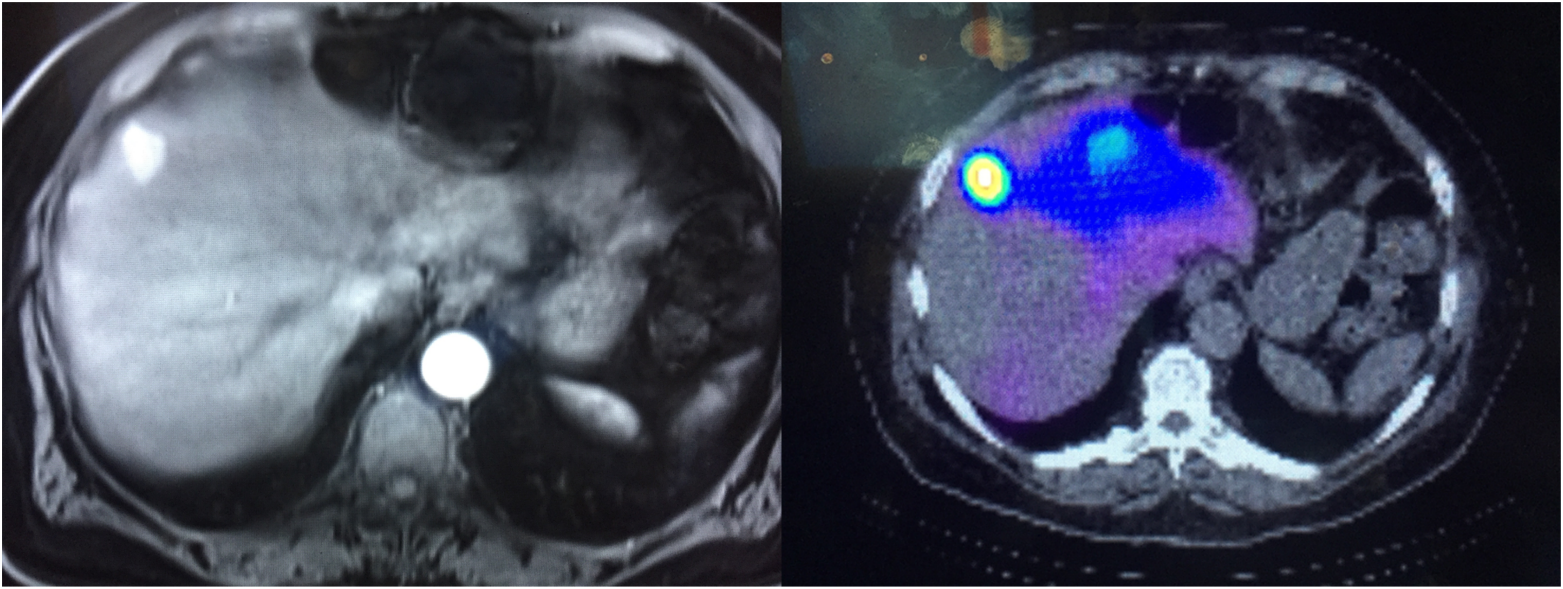
Left shows a contrast-enhanced CT of the renal cell carcinoma lesion being treated. Right shows a post-therapy fused non-contrast CT and bremmstrahlung SPECT.

Y-90 radioembolization techniques are now a standard tool for the treatment of primary liver cancer and liver-dominant metastatic disease (10). These procedures involve the use of micron-sized glass or resin spheres that are infused directly into the patient near the site of disease where capillary action embeds the spheres into the tumor bed. The spheres deliver a high beta radiation dose to the tumor site resulting in effective, targeted treatment. A metastatic renal cell carcinoma therapy example is shown in Figure 1.

Only two devices are approved for radioembolization therapy so the procedures associated with their use are fairly standardized even though international and interdepartmental differences can be observed. What has been published less over the years are nuanced differences between delivery techniques and handling of the approved products. Understanding the commonalities and differences between these therapies can assist with more informed handling of these products as well as improve the training of physicians and staff.

### 1.1. Therapy product overview

Two commercially available and FDA-approved radioembolization devices were compared throughout the study to assess nuanced, but key differences in therapy delivery, handling of the products, and radiation exposure of patients and staff between the available products. SIR-Spheres is a resin microsphere that is molecularly bound to Y90, while TheraSpheres is a glass microsphere made from zirconium and bombarded with neutrons in a reactor to create a yttrium-90 glass matrix. Both products are used similarly, however, TheraSpheres are specifically indicated for the treatment of unresectable HCC while SIR-spheres are indicated for the treatment of unresectable metastatic liver tumors from primary colorectal cancer (11).

TheraSpheres are generally smaller in average diameter than SIR-spheres with diameters ranging from 20–30 μm vs. 20–60 μm. Activity per bead is also higher with TheraSpheres with typical activity ranges of 2,400–2,700 Bq per bead (40–70 Bq/bead for SIR) (12,13). Both products are delivered in dose vials, however, TheraSpheres vials are ordered based on calculated doses for the day of delivery while SIR-spheres doses are sent in standard vial sizes from which the patient dose is extracted on the day of the procedure by the nuclear medicine or pharmacy staff.

## 2. Methods

This study is broken into two primary sections. The first focuses on differences between delivery techniques and methods for each product and the second examines radiation safety and handling characteristics of each device. The research was performed under the auspices of a University of Tennessee Graduate School of Medicine Institutional Review Board-approved protocol (IRB# 4388). Patients were recruited through our University of Tennessee Medical Center interventional radiology clinic from those individuals being referred for Y90 radioembolization therapy for HCC or metastatic liver disease.

### 2.1. Therapy delivery

**Figure 2:**
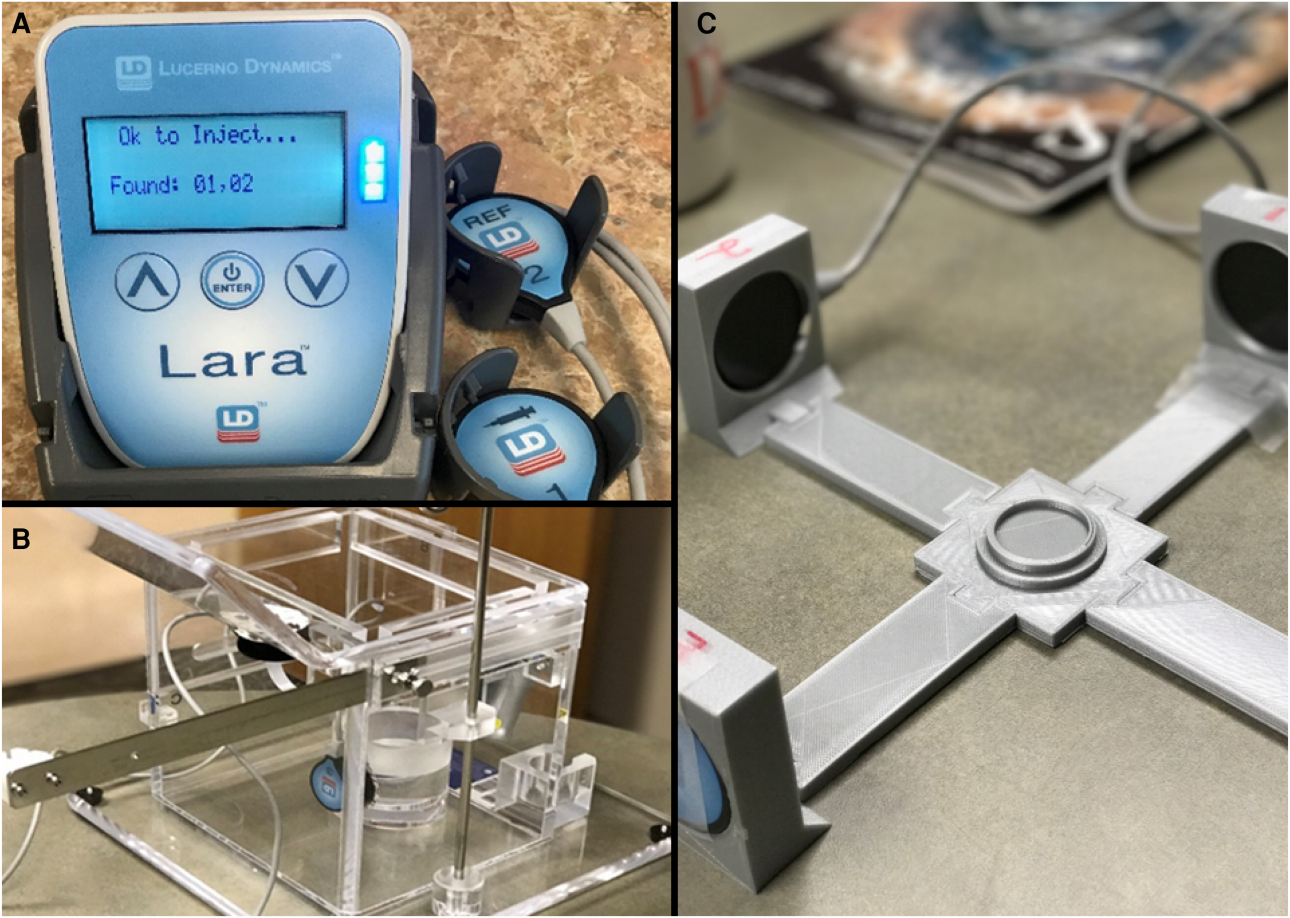
(**A**) Shows the detector system with readout, (**B**) shows the detector mounted inside of the radioembolization dose delivery system housing, and (**C**) shows the quality control mechanism prior to research use.

Therapy delivery characteristics were assessed by using an external radiation detector system (Lucerno Dynamics, Cary, NC) that uses modular detectors connected to a readout where results can be uploaded and displayed. This system is an FDA listed device for injection monitoring with which our group has extensive operational experience having used these systems for nearly ten years and published many conference abstracts and peer-reviewed publications using these devices (14–18). The platform also includes a real-time readout of the count rate during acquisition (Figure 2A). Detectors were placed in four different locations for each of the therapy products inside of each delivery apparatus facing the dose vial and on the output catheter lines to the patient (Figure 2B). Additional detectors were placed near the liver of the patient and on top of the foot to measure extremities. Detector data were acquired continuously throughout therapy delivery to collect time activity curves (TACs) related to each procedure. These data were analyzed to try and determine the characteristics of each therapy procedure type to see if additional insights into how these therapies are delivered could be found and to potentially improve in-room delivery procedures.

Quality control (QC) was performed prior to detector usage for monitoring patient procedures. Detectors were placed into a custom calibration system designed by our team (Figure 2C). The dose for the patient was placed into a central holder mechanism with the detectors placed into mounts that placed each one equidistant from the dose (see figure). Data were collected for several minutes to determine count rates from the full dose. The final curves were used to determine any changes in detector performance that might need correction prior to patient usage. QC records were uploaded to online storage for later comparison.

#### 2.1.1. On-table assessment of infusion quality and residual dose

The first characterization performed was assessing whether monitoring could provide additional information regarding the quality of the infusion and residual dose. Currently, injection quality is only assessed using residual doses which are only measured after the procedure has been completed and the patient removed from the room. Additionally, there are no good feedback mechanisms in the procedure room that enable the treatment staff to get confirmation that the dose was injected without issue. Although delivery issues such as major system leaks require aborting the dose delivery, other delivery issues such as a temporarily blocked lines, some stasis occurrences, and external leaks resulting in insufficient pressure can be mechanically resolved.

Fifteen patient procedures (8 Theraspheres/7 SIR-Spheres) had external radiation sensors placed onto the respective radioembolization delivery system with one sensor placed approximately two inches from the dose vial and the other sensor placed directly on the delivery line to the patient. The detectors were connected to a digital reader that collected the signals from the detector during the course of therapy delivery. Following infusion completion, the data from the reader module was uploaded and reviewed in near real-time to assess the injection. The count rate curves were then further analyzed to determine if accurate estimations of residual activity could be made prior to the patient leaving the procedure room and to determine how the in-room estimates compared to the gold standard measurements performed in Nuclear Medicine.

**Figure 3:**
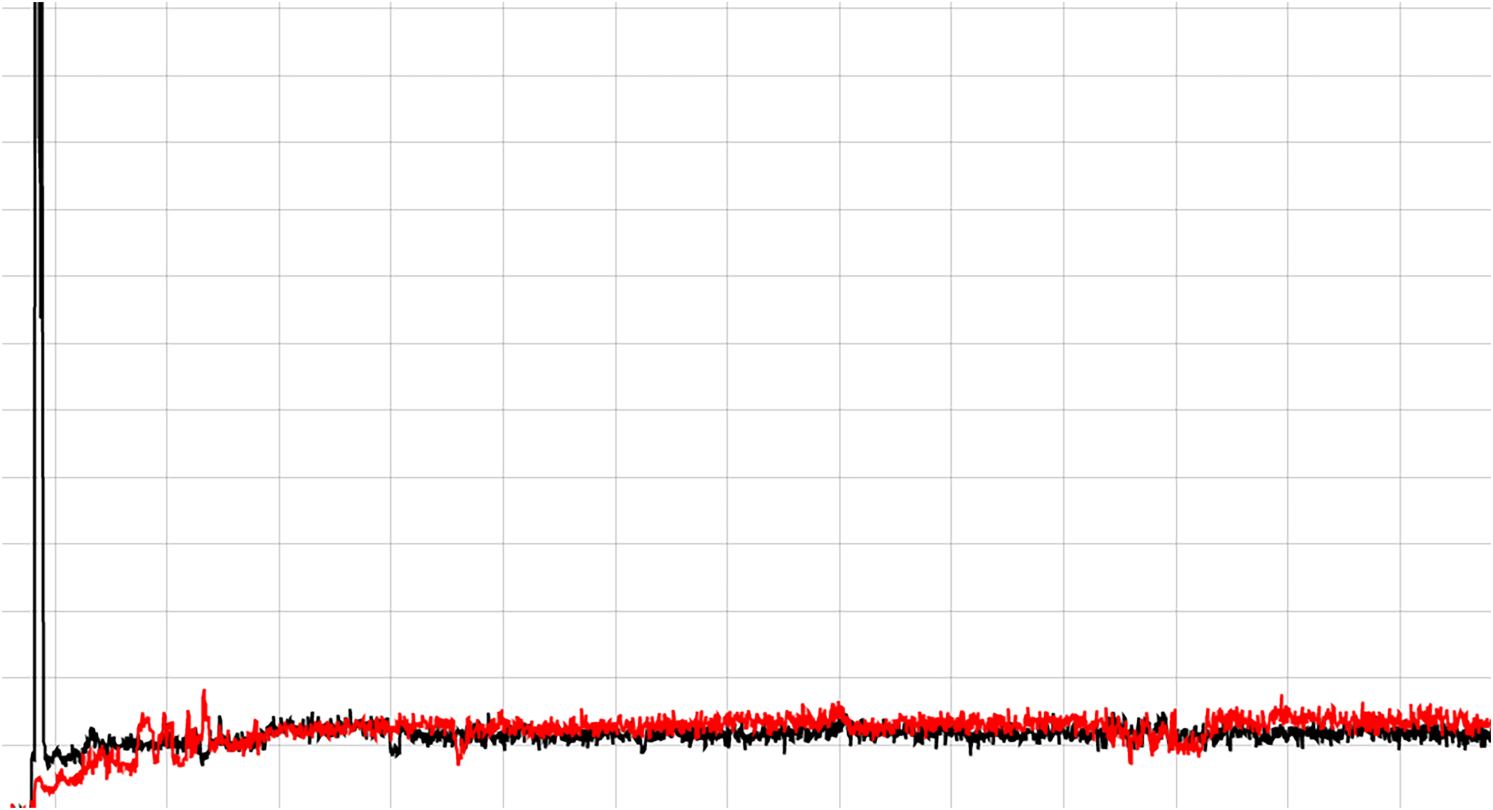
Shows a good quality PET/CT radiopharmaceutical injection. The black line is the detector distal to the injection site and the red line is a reference location on the opposite arm. A good injection is indicated when the radiation detected across the detector increases rapidly as the dose passes by and then quickly falls back to baseline.

For in-room assessments of injection quality and residual dose, the uploaded data were visualized using the system’s web interface tools. This enables a quick visual review and quantitative assessment of count rate information collected. Visual analysis parameters used to describe a good quality injection were if the count rate levels following infusion were very low compared to peak values and if a drop in activity could be visualized during infusion that showed a smooth, progressive infusion similar to how we assess routine diagnostic injection quality (19–21). An example of a typical time activity curve (TAC) for a good quality radiopharmaceutical injection for a standard nuclear medicine tracer is shown in Figure 3 to highlight how the real-time monitoring functions. This image will also help highlight the unique differences in the microsphere therapy delivery curves compared to a typical radiopharmaceutical injection.

The analysis workflow varied depending on whether glass or resin sphere radioembolizations were used described in detail for each below. For resin beads, the gold standard for residual dose assessment is to measure the dose vial prior to infusion and after the infusion is complete (22). The ratio of those values is calculated as a percentage and recorded on the patient’s written directive. Estimates were obtained from the time activity curves from the monitoring device by placing the cursor of the web tool on areas of peak activity seen when the dose first passed through the input catheter and the value recorded. The cursor was also placed at the location where the dose can be visualized to have been the last flush of the dose and that lower value was recorded. The residual dose percentage was inferred by the percentage of peak count rate to post-infusion count rates.

For glass beads, the clinical methodology for determining residual activity is based on exposure rate measurements of the initial dose on a template compared to dose rate measurements of all waste materials following infusion (22). The exposure rate values are scaled to activity and the ratio of pre and post-infusion values are used as the residual. As the infusion of glass beads appears as a bolus with the external monitoring system, a different calculation method was employed to estimate residual percentages. Time activity curves were characterized by calculating the “knee” of the curve to serve as a baseline for the lower point of the injection. The integral of the peak value to “knee” (total counts) was used as a measure of total activity over a relative period of time. The ratio of the activity at the knee to the total counts was calculated to provide an estimate of residual dose. In this framework, the “knee” value characterizes how low the measured count rate got to the baseline and the integration of the peak to baseline measure characterizes the maximum count rate measured relative to the rate at which the count rate dropped as the measurement approaches the baseline.

(1) $$\text{Residual Resin Spheres \%}=\frac{\text{post-infusion count rate}}{\text{peak count rate}}\times 100$$

(2) $$\text{Residual Glass Spheres \%}=\frac{\text{"knee" count rate value}}{\text{total counts}}\times 100$$

The time activity curves generated from each patient measurement were downloaded into standard spreadsheet software for further comparison to nuclear medicine estimates of residual dose. Standard post-procedure assessments of residual activity are performed by either measuring the vial in a dose calibrator (SIR-Spheres) or by measurement of all waste elements by ion chamber (TheraSpheres). Residual estimates from the monitoring device, as noted above, were used to calculate percent differences between device estimates and nuclear medicine measurements with a standard t-test used to assess any statistical difference between the methods for residual calculation for each bead type.

### 2.2. Radiation safety and handling

Radiation safety aspects were assessed by monitoring radiation exposure to those staff involved in handling each of the available therapy products from initial manipulation to completion of therapy delivery. Nuclear medicine technologists were given ring dosimeters for each hand as well as a dosimeter for determining body exposure with each badge used by that technologist for a total of five different procedures to get the average dose per procedure estimates. Ring badges and body dosimeters were worn from the time the radiation package was opened through the technologist’s final interaction with the dose. Interventional radiology physician body and hand doses were measured for each delivered therapy using standard body and ring dosimeters.

Handling variations between the two radioembolization products were assessed by evaluating our standard clinical process for each process. Steps for dose preparation and administration were analyzed and compared with times recorded for critical processes, including average dose preparation time by the technologist, average time of dose delivery by the physician, and the average time for technologists in interventional radiology to perform surveys on staff and the room. Handling times were compared for each radioembolization device.

To characterize uncommon and little-studied aspects of radiation safety related to radioembolization, a three-phase assessment of off-target and potentially leached Y90 was performed to assess for any “free” and aggregate Y90 in the patient. First, the TACs collected from the monitoring device placed over the top of the foot were used to visualize radioactivity to assess for high count rates in the extremities that would be indicative of significant off-target activity. Then, blood samples were collected before, during, post infusion, and just before follow-up PET/CT imaging (typically 2–4 h post-therapy). Blood samples were counted on a gamma counter to determine activity in the blood at each time point. Finally, each patient in this study received a post-treatment whole-body PET/CT covering an axial extent of the base of the skull to mid-thigh to assess for any significant aggregate Y90 deposition that may have resulted from circulating free Y90 in the subject.

PET/CT imaging of the patients was performed on our 64-slice Biograph mCT (Siemens Healthineers, Knoxville, TN). Data were acquired using a continuous bed motion protocol over an axial extent covering at least the base of the skull to mid-thigh. Data for each patient were acquired for approximately 45 min with subsequent histogramming and reconstruction that incorporated time of flight and used ordered subset maximization expectation (OSEM)-based point spread function algorithms.

Image analysis of whole-body PET/CT images was performed using the Inveon Research Workplace (Siemens Healthineers, Knoxville, TN) and Radiant DICOM Viewer (23) (https://www.radiantviewer.com). Data were visualized using multi-planar reconstruction to examine all dimensions. Maximum intensity projections (MIPs) were created and various thresholds used to examine for visible signs of significant PET signal outside of the target therapy region with a specific focus on examination in critical organs and extremities. Additional data processing was also used, including the summation of slices and various image filters.

## 3. Results

### 3.1. Infusion quality and residual dose

**Figure 4:**
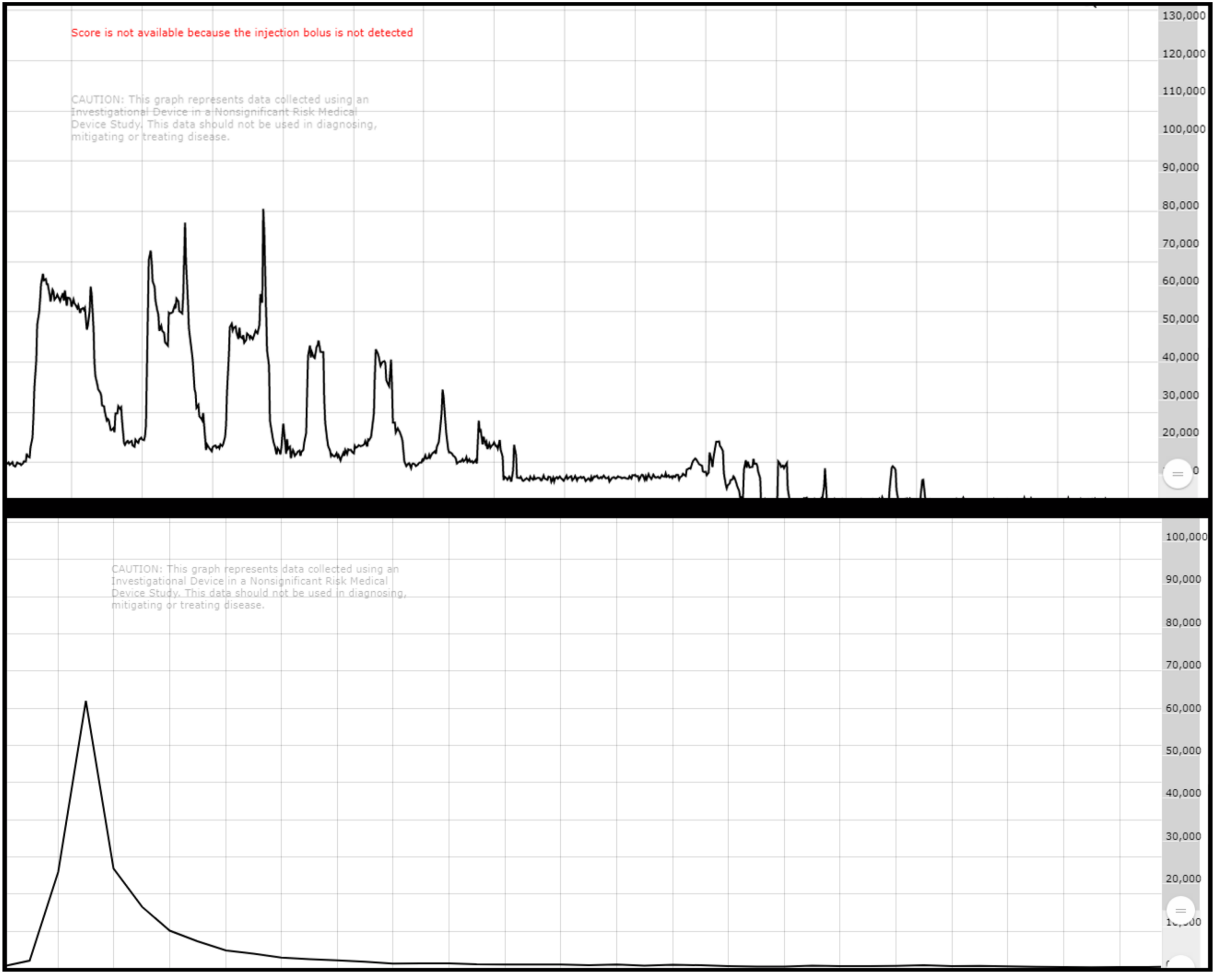
Top shows a characteristic SIR-spheres infusion with the puff/fluoro technique showing decreases in activity through the catheter as the dose is pushed from the vial. Bottom shows a characteristic Theraspheres infusion with a bolus injection appearance.

Visual inspection of infusion quality showed characteristic injection patterns between SIR-spheres and TheraSpheres delivery methods. The “puff/fluoro” routine used for SIR-spheres showed clear patterns of dose moving through tubing followed by fluoroscopic pulses with each push of spheres resulting in a smaller and smaller count rate value until reaching baseline values. TheraSpheres visual assessments show a rapid bolus with the peak activity passing quickly and a rapid fall to baseline values. The example curves shown in Figure 4 demonstrate that for each case study in this work the count rate values returned to baseline following administration of the microspheres.

Residual dose estimates by nuclear medicine were less than 6.5% in both glass and resin sphere radioembolization procedures examined in this study (Table 1). Comparison of residual estimates made using the external monitoring device differed from gold standard nuclear medicine calculations by an average of 2.2% and 1.9% for TheraSpheres and SIR-Spheres, respectively, and was not found to be statistically significant (p>0.05). Reduced variability in residual dose estimates was observed from the measurements made using the detector system with a reduction in coefficient of variation (COV) of greater than 33% for both microsphere therapies compared to gold standard nuclear medicine assessments. COVs for the nuclear medicine measurements that are the clinical standard were 0.85 and 0.55 for TheraSpheres and SIR-Spheres, respectively.

**Table 1:** Comparison of residual dose measurements between gold standard nuclear medicine calculations and the external detection system.

Gold standard residual vs. In-room residual estimate	
% Theraspheres difference	% SIRSpheres difference
0.11%	1.40%
3.65%	3.12%
4.41%	0.25%
1.23%	1.83%
3.62%	3.64%
1.72%	0.99%
3.90%	1.83%
0.14%	
0.94%	
**Average difference**	
Therasphere	SIR-sphere
2.2%	1.9%

### 3.2. Radiation safety and handling

Radiation safety assessments showed significantly greater dose to the hands of technologists using SIR-Spheres compared to TheraSpheres. Physician body and hand doses were negligible for both microsphere therapies, however, a statistically significant decrease in dose was nevertheless observed for TheraSpheres compared to SIR-Spheres usage. For both radioembolization procedures, radiation doses to all staff involved in preparation and delivery were well within safe handling limits.

**Figure 5:**
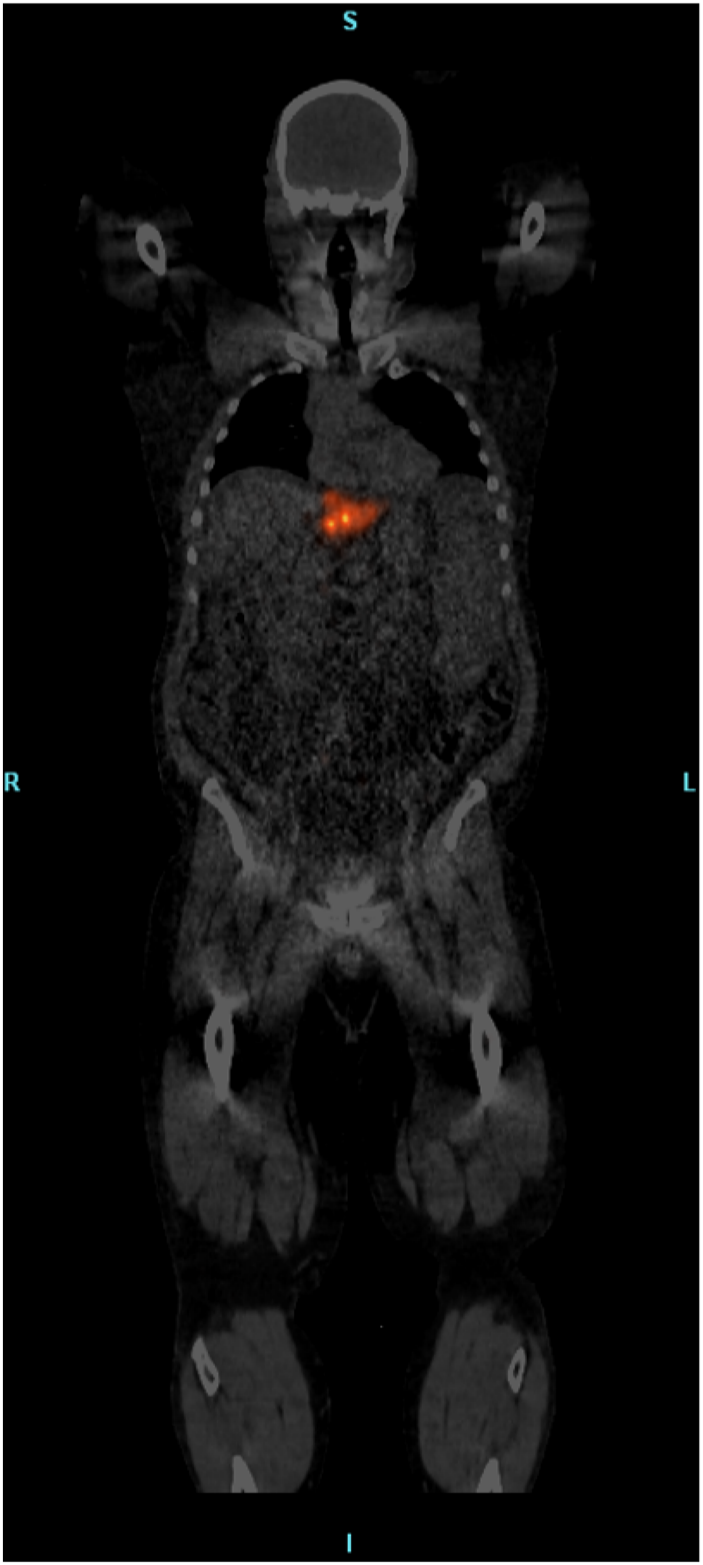
Whole-body fused PET/CT image showing only focal activity at the site of treatment and no widespread aggregation of free Y90 detected.

Blood samples acquired from patients showed statistically significant increases in free Y90 blood radioactivity immediately following completion of SIR-Spheres administration with a 120% increase compared to blood taken pre-infusion. TheraSpheres blood activity showed a small increase of 7% between pre-administration and during infusion samples, however, the radioactivity continually declined throughout the administration. COVs for glass microsphere’s blood activity levels were much lower at 0.008 versus 0.45 for resin sphere blood activity. For both products, the free Y90 radioactivity levels in the blood were at pre-administration levels by the time of PET/CT imaging (approximately 2–4 h post-infusion). Whole-body PET/CT imaging did not show any aggregate Y90 for SIR-Spheres or TheraSpheres patients outside of areas where slight off-target activity could be expected (lungs, bladder, etc.). Even with the use of summed slice assessments, maximum intensity projections, and various image filtering, no appreciable extremity or other organ activity was observed using either device with an example whole-body PET/CT image shown in Figure 5.

Procedure timing comparisons at our facility between the two devices indicated a fourteen-step process for SIR-Spheres and a four-step process for TheraSpheres. The handling times for the three categories tested were, on average, approximately 30% lower for TheraSpheres than SIR-Spheres with dose preparation and delivery times being approximately 50% lower. The average time for the technologists in the room was nearly identical for both devices averaging approximately 35 min.

## 4. Discussion

This study sought to examine a wide range of unique aspects of radioembolization therapies, but is limited in the number of patients on which analysis was performed. Although some statistically significant values were found, the small number of patients used in the study limits the generalization to large populations of radioembolization cases. A larger patient population testing residual dose estimates and applicability to resident and professional training is needed to further validate the efficacy.

The whole-body PET/CT imaging protocol used in this study was designed to be fully quantitative and data acquisition times set to yield the best possible results in a reasonable time frame. The published information regarding leaching of Y90 from resin microspheres indicates low levels of unbound material, (0.4% to a specified maximum expected of 5%), however, Y90 is known to easily bind to structures (24,25). Although these amounts are quite low overall, aggregation of the free Y90 to off-target sites over a possible series of infusions may not be inconsequential.

The post-therapy imaging portion of our study was to acquire whole-body images to determine if any of the free-circulating Y90 measured from whole blood radiation measurements resulted in any detectable aggregations of activity in off-target sites. No aggregate Y90 was visualized at the 2–4 h post-infusion imaging, which parallels our blood activity measurements at the time of PET/CT imaging indicating pre-administration activity levels. One weakness to any imaging method activity levels were simply too low for detection using 3D PET. Although sensitivity for low activity when trying to image Y90 on a PET system is a concern, the correlated blood radioactivity levels showing a return to baseline for both products helps to bolster the negative findings on PET/CT imaging.

In this study we also introduce a novel methodology for real-time monitoring of therapy delivery and infusion characteristics. These monitoring activities do not impact the therapy workflow negatively and can provide additional insight into the status of the delivery. Active monitoring of the infusion potentially enables staff to intervene should something go wrong during administration of the therapy. Such interventions can include, mechanical release of tubing that becomes pinched during infusion, loss of infusion pressure, etc. Residual dose measurements in the room can also be estimated which could be helpful in estimation of the impact of any infusion issues that occurred during infusion or to possibly adjust subsequent infusions for multiple dose cases. Because these TACs are stored and accessible online, these can be analyzed following a procedure to examine technique and inform the physician on their overall infusion quality. This analysis can also be used to educate other physicians and residents regarding their delivery technique of infusions that could enable optimization of therapy deliveries and provide more consistent procedure training.

Radiation safety concerns vary depending upon whether resin or glass spheres are used. Although neither of the products showed build-up of free Y90 in the patients, resin spheres showed significantly more circulating Y90 in the blood which eventually will be cleared by the renal system and excreted. This requires sites to ensure they have appropriate release criteria and instructions for their patients undergoing resin sphere radioembolization. Sites using resin spheres will also have staff that will be handling the product for longer periods of time resulting in increased extremity exposures, however, since body exposures were similar across both radioembolization products, sites can easily optimize their handling procedures using ALARA concepts to limit the additional exposure to their staff from resin microsphere handling.

## 5. Conclusion

Radioembolization using microspheres is a common therapeutic procedure, however, some critical aspects related to dose delivery and handling have not been studied thoroughly. New insights into Y90 radioembolization techniques can be gleaned through the use of real-time monitoring systems to measure time activity curves during device infusion that can improve the quality of treatment and assist with staff education. Radiation safety aspects differ between radioembolization products and facilities should be aware of these differences to limit radiation exposure to staff administering these therapies.

## Data Availability

The datasets generated for this study are not publicly available due to the HIPAA protected nature of the data. Requests to access the datasets should be sent to the corresponding author.
